# Obstacles to researching the researchers: A case study of the ethical challenges of undertaking methodological research investigating the reporting of randomised controlled trials

**DOI:** 10.1186/1745-6215-11-28

**Published:** 2010-03-21

**Authors:** Joanne E McKenzie, G Peter Herbison, Paul Roth, Charlotte Paul

**Affiliations:** 1 School of Public Health and Preventive Medicine, Monash University, Melbourne, Australia; 2 Department of Preventive and Social Medicine, University of Otago, Dunedin, New Zealand; 3 Faculty of Law, University of Otago, Dunedin, New Zealand

## Abstract

**Background:**

Recent cohort studies of randomised controlled trials have provided evidence of within-study selective reporting bias; where statistically significant outcomes are more likely to be more completely reported compared to non-significant outcomes. Bias resulting from selective reporting can impact on meta-analyses, influencing the conclusions of systematic reviews, and in turn, evidence based clinical practice guidelines.

In 2006 we received funding to investigate if there was evidence of within-study selective reporting in a cohort of RCTs submitted to New Zealand Regional Ethics Committees in 1998/99. This research involved accessing ethics applications, their amendments and annual reports, and comparing these with corresponding publications. We did not plan to obtain informed consent from trialists to view their ethics applications for practical and scientific reasons.

In November 2006 we sought ethical approval to undertake the research from our institutional ethics committee. The Committee declined our application on the grounds that we were not obtaining informed consent from the trialists to view their ethics application. This initiated a seventeen month process to obtain ethical approval. This publication outlines what we planned to do, the issues we encountered, discusses the legal and ethical issues, and presents some potential solutions.

**Discussion and conclusion:**

Methodological research such as this has the potential for public benefit and there is little or no harm for the participants (trialists) in undertaking it. Further, in New Zealand, there is freedom of information legislation, which in this circumstance, unambiguously provided rights of access and use of the information in the ethics applications. The decision of our institutional ethics committee defeated this right and did not recognise the nature of this observational research.

Methodological research, such as this, can be used to develop processes to improve quality in research reporting. Recognition of the potential benefit of this research in the broader research community, and those who sit on ethics committees, is perhaps needed. In addition, changes to the ethical review process which involve separation between those who review proposals to undertake methodological research using ethics applications, and those with responsibility for reviewing ethics applications for trials, should be considered. Finally, we contend that the research community could benefit from quality improvement approaches used in allied sectors.

## Introduction

A well conducted randomised controlled trial (RCT) is the most reliable method for measuring the effectiveness of a clinical intervention. When properly implemented, many of the biases observed in non randomised and observational studies can be removed [1]. However, the benefits of the RCT study design are only realised when they are properly implemented and reported.

There is a large body of literature, across many disciplines, suggesting that current conduct and reporting of many RCTs is inadequate (examples include [2-6]). Inadequate conduct and reporting of RCTs has been associated with bias in estimating intervention effects [7-10]. For one component, inadequate allocation concealment, this bias has been estimated as a 30% exaggeration in intervention effect [7]. Other forms of bias can occur from selective reporting of results. This can occur when there is selective reporting of either the entire RCT (between-study selective reporting bias or publication bias), or of the results within a RCT (within-study selective reporting bias).

Much research has been undertaken investigating between-study selective reporting bias, but relatively little investigating within-study selective reporting bias. However, some recent studies investigating the latter have highlighted some concerning results [11-17]. Two landmark studies investigated the prevalence of unreported outcomes by comparing study trial protocols with subsequent trial publications. They estimated that for efficacy outcomes, 71% (95% confidence interval (CI): 61%, 79%) and 88% (95%CI: 75%, 95%) of RCTs had at least one unreported outcome [12,13]. These studies estimated that statistically significant outcomes had a higher odds of being fully reported compared with non-significant outcomes for both efficacy and harm outcomes; where fully reported was defined as outcomes with sufficient data for inclusion in a meta-analysis. For efficacy outcomes the odds ratios ranged from 2.4 (95%CI: 1.4, 4.0) to 2.7 (95%CI: 1.5, 5.0) while for harm outcomes the range was from 4.7 (95%CI: 1.8, 12.0) to 7.7 (95%CI: 0.5, 111). A systematic review summarising the evidence from empirical studies investigating publication bias and within-study selective reporting bias concluded for the latter that statistically significant outcomes were more likely to be more completely reported compared to non-significant outcomes [18]. Four studies, including those above, contributed to this conclusion.

In addition to misrepresenting the results from the RCT, bias arising from selective reporting can impact on meta-analyses [14], influencing the conclusions of systematic reviews, and in turn, evidence based clinical practice guidelines [19-21]. This will, in some instances, result in wrong answers to important clinical questions. Patients, clinicians, and health policy makers rely on this information for evidence based decision making. Researchers and grant funding bodies rely on this information to inform future research including the need for future RCTs [21,22].

There has been no published research in New Zealand (NZ) investigating within-study selective reporting bias. Awareness of the quality of reporting of RCTs is a first step in determining if there is a need to develop processes to ensure adequate reporting of future RCTs. In this publication we report on a methodological research project we planned to undertake to investigate the quality of reporting of RCTs in NZ, including within-study selective reporting bias. We discuss the ethical challenges we faced in trying to undertake this research and discuss potential solutions.

## What did we plan to do?

Two of the authors of this publication (JEM, GPH) attempted to undertake a study similar to Chan *et al'*s [12] study to investigate within-study selective reporting bias of outcomes in a cohort of RCTs submitted to New Zealand Regional Ethics Committees (NZRECs) in 1998/99, where the trial was based in NZ. Additional aims of the study included: (i) describing the process and outcome of the cohort of RCTs, (ii) assessing the adequacy of reporting of RCTs and identifying barriers to adequate reporting, and (iii) comparing the consistency of ethics applications with corresponding published articles in terms of: primary outcome(s), secondary outcome(s), sample size, methods of allocation concealment used, and blinding. We were successful in receiving an internal university grant (University of Otago Research Grant), through a competitive grant process, in October 2006, which provided one years funding.

In brief, the study involved identifying and accessing ethics applications of approved RCTs submitted to NZRECs in 1998/99, along with their amendments and annual reports. In NZ there is no requirement to submit a full trial protocol as part of the ethics application and so the information contained in the ethics applications is limited and variable. However, despite this, they still provide the best source of information to evaluate the completeness of RCT reporting in NZ. We chose the years 1998/99 to provide a reasonable length of time for the RCTs to be carried out, written up, and published [23]. A literature search of relevant databases would then be carried out to identify publications of the included RCTs. Data would be extracted from the ethics applications, annual reports, amendments, and publications. Two questionnaires would be sent to trialists. In the first questionnaire, trialists would be provided with a list of unreported outcomes which had been identified from comparison of the ethics application and published article. The statistical significance of unreported outcomes and reasons for omitting them would be solicited. A second questionnaire would assess trialists' knowledge of the CONsolidated Standards Of Reporting Trials (CONSORT) statement, a guideline for reporting RCTs, and barriers to its use [24].

To identify ethics applications for inclusion, we planned to screen full ethics applications submitted to NZRECs in 1998/99. This was necessary since it is often not possible to identify which applications are RCTs from information contained in the NZRECs' annual reports.

We planned to actively seek the participation of trialists to complete the questionnaires. However, for practical and scientific reasons, we did not plan to obtain consent from trialists to access their ethics application. Practically, if we were only able to use NZREC annual reports to identify ethics applications, we would be limited to the title of the ethics application and the name of the primary investigator. Finding contact details of all the potential investigators would be difficult given the age of the records. Over these two years, we estimated that in excess of 1500 applications were submitted to NZRECs for consideration. In addition, we were concerned that the proportion of investigators who would provide consent for us to access their ethics applications would be low [25]; providing little yield for a large effort. Moreover, it could prejudice the scientific value of the study since trialists with less exacting reporting practices might be less likely to allow us access to their ethics applications, thus biasing and undermining the results of the study.

Moreover, we believed that there was minimal risk to trialists by us undertaking this study. Two issues which we considered trialists may have concerns about were identification of defects in their study reporting which could be used to disadvantage them, and disclosure of commercial and intellectual secrets from their ethics application. In regard to both issues, only the two researchers on the grant (JEM, GPH) and their research assistant would view the ethics application. Information from the ethics applications would be treated confidentially, and importantly, in publications arising from our research we would present the results using summary statistics on groups of ethics applications/publications. Therefore, studies would be highly unlikely to be identifiable, thus protecting the anonymity of the trialists.

Finally, ethics applications are official information held by the NZ public sector and therefore access to them is governed through the Official Information Act 1982. Under this act, information must be made available when requested unless good reason exists for it to be withheld. It is the ethics committee's responsibility to make applicants aware of this [26].

## What issues did we encounter?

**Figure 1:**
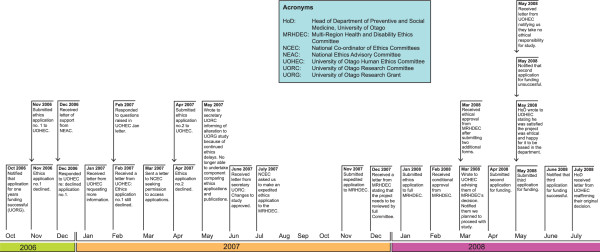
**Project history of "Conduct and reporting of randomised controlled trials in New Zealand"**.

**Figure 2:**
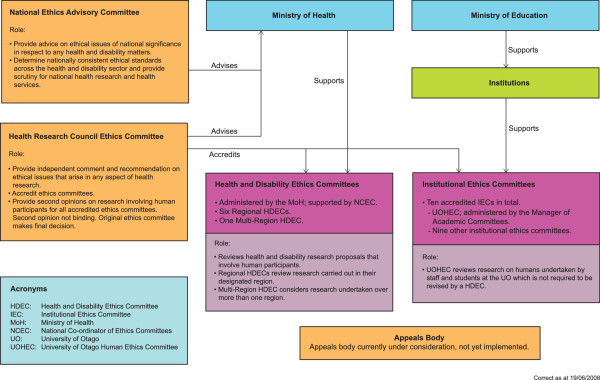
**New Zealand National System of Ethics Review**.

The timeline for the project is outlined in Figure 1. In November 2006 we sought ethical approval for the project from our institutional ethics committee, the University of Otago Human Ethics Committee. This ethics committee is accredited by the Health Research Council Ethics Committee and is one of a number of committees in NZ which forms part of a national system of ethics review (Figure 2) [27]. Our institutional ethics committee has two categories of application; category A applications are considered by the Committee, while category B applications are for low-risk research involving human participants, and are assessed at the Departmental level. While our project did not meet the criteria for category A, we made a decision to submit it as such, so that it would be considered by the full Committee, because we felt there were sensitivities involved in accessing ethics applications without gaining informed consent from trialists to do so.

The ethics application was declined by our institutional ethics committee in November 2006, and this initiated three months of communication with the Committee (Figure 1). The primary issue raised by the Committee was informed consent.

"The application states that the "participants" for the study are "investigators who submit applications for approval for randomised controlled trials that are published at the time of the search". A fundamental principle of ethical research is, of course, informed consent. The Committee is concerned that "participants" are not informed prior to the research. They will not be exercising voluntary choice to participate nor will they be informed of the purpose, methods, risks, inconvenience and possible outcomes, including publication of the research. Also, investigators who have submitted applications to relevant ethics Committees have not agreed to participate in such a project as part of their application process." (*Personal communication: manager, Academic Committees, University of Otago, 21 November 2006*)

The Committee also questioned "... the relevance of the research given the advent of clinical trial registers and is of the view that such oversight as that proposed in the application may be obsolete." (*Personal communication: manager, Academic Committees, University of Otago, 21 November 2006*). They perceived, and did not approve of us "becoming self-appointed auditors", and felt that a project such as this would "... more appropriately be undertaken as an audit by the relevant ethics committees" (*Personal communication: manager, Academic Committees, University of Otago, 21 November 2006*). They requested clarification of the data to be extracted from the ethics application (*Personal communication: manager, Academic Committees, University of Otago, 19 January 2007*). They questioned "... whether applications for ethical approval that have been submitted to an ethics committee are in the public domain" and questioned "Will authors be surprised to be contacted about information that they have provided in their ethics applications?" (*Personal communication: manager, Academic Committees, University of Otago, 19 January 2007*).

In consultation with researchers with expertise in ethical and legal issues (CP, PR), we responded to our institutional ethics committee outlining legal and ethical reasons for why we considered it was reasonable to undertake this research without obtaining informed consent. In brief, we provided legal justification for accessing the ethics applications with reference to the two NZ statutes that deal with freedom of information, the Official Information Act 1982 and the Privacy Act 1993. The former provided every reason why the information sought ought to be disclosed, and the latter provided no justification for it to be withheld or not used. The legal presumption was that the information concerned ought to be made available. There was no legal impediment standing in the way. Indeed, the public policy reasons underlying the enactment of freedom of information legislation would have been satisfied had the information been made available for the purpose of holding both trialists and ethics committees publicly accountable. Details of the legal issues are available in Additional file 1 - What are the legal issues?

We provided ethical justification for undertaking the project without obtaining informed consent from the trialists to access their ethics applications, making reference to the Ethical Guidelines for Observational Studies [28]. This document, prepared by the National Ethics Advisory Committee (NEAC), aims to provide guidance to researchers and ethics committees for carrying out observational research. It proposes that access to personal health information without consent is justifiable when seeking consent would not be practicable or would affect the scientific value of the study; and when there would be no disadvantage to the participants; and there is a public interest in the study.

We provided clarification of the data to be extracted from the ethics applications, justification for undertaking the research with the advent of clinical trial registers, and reasons for why we considered we were an appropriate group to do this. While registration of clinical trials is a positive step to improve reporting, we believed, and conveyed to our institutional ethics committee, that this alone was unlikely to be the only solution since ethics committees in NZ do not insist on registration as a requirement for ethical approval, only a few journals insist on pre-registration as a condition of publication, and currently there is no requirement to register all outcomes in a trial. We responded to the latter criticism by stating that ethics committees in NZ generally have neither the resources nor the expertise to undertake such studies and that researchers have carried out such studies previously. Further, the authors of this study (JEM, GPH) have methodological and statistical expertise consistent with researchers who have carried out previous studies. In addition to appropriate expertise, we stated that independence from the ethics committee process should be considered a strength. We responded to the question of "surprise" by stating that trialists may be surprised when contacted, but they would be provided with an explanation about the project and the rationale for accessing their ethics application, and would have the option of not completing the questionnaire. Finally, we provided our institutional ethics committee with a letter of support which we had received from the NEAC after advising them of our study (Figure 2). NEAC considered that this study would add valuable NZ dimensions to the reporting of RCT results.

After three months of communication, and consideration of all the information supplied, our institutional ethics committee declined the application. The Committee did not accept that the principal investigators needed to be contacted (i.e. to complete the questionnaires) and believed that "the research would continue to be valid if this part was modified" (*Personal communication: manager, Academic Committees, University of Otago, 27 February 2007*). They stated that if we wished to undertake the study without making contact with the principal investigators, we should submit an amended application for ethical consideration. Our institutional ethics committee also regarded the use of the Official Information and Privacy Acts as "... unduly aggressive" (*Personal communication: manager, Academic Committees, University of Otago, 27 February 2007*).

**Figure 3:**
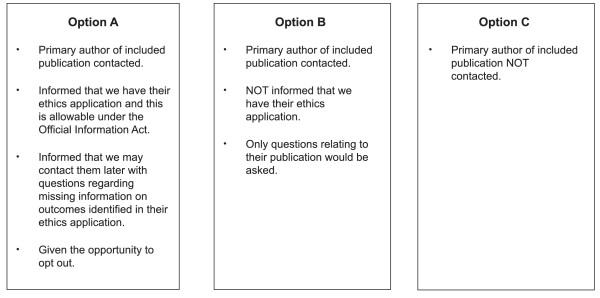
**Three proposed options to undertake the study**.

A second ethics application was submitted in April 2007 (Figure 1). We presented three options which we believed addressed the concerns raised by our institutional ethics committee in various ways (Figure 3). We considered option A (which was similar to our original ethics application) to be the most ethical and scientifically tenable. It provided the most transparency since trialists were informed of the study and that we had a copy of their ethics application. Scientifically, it was more likely to provide complete data on outcomes not reported in publications and provide trialists with an opportunity to explain discrepancies between their ethics application and publication(s), which may be legitimate [29].

The second application was also declined on grounds of informed consent not being obtained from trialists before retrieving their ethics applications.

"The Human Ethics committee has construed that the "primary authors" of publications to be the participants in this project. A guiding ethical principle of the Committee is for researchers to respect the welfare, rights, beliefs and perceptions of participants. In view of the Human Ethics Committee it is the researcher's responsibility to minimise risks of harm or discomfort in the participants and most importantly research protocols should be designed to ensure the respect, dignity and well being of the participants. A fundamental principle of ethical clearance is that participation is voluntary and that informed consent must be provided before individuals participate in the project." (*Manager, Academic Committees, University of Otago, 27 April 2007*)

However, the ruling was somewhat ambiguous. The Committee stated that while we were legally able to undertake the 'audit' component of the project without the need for ethical clearance, they could not approve the process. They had particular concerns surrounding us contacting trialists, and concluded with the following: "In particular, the second stage, involving a response to questionnaires, cannot proceed without ethical approval. It is possible, however, for you to conduct this first 'audit' stage without the Committee's approval, and to submit an application to complete the second stage once you have identified the potential participants." (*Personal communication: manager, Academic Committees, University of Otago, 27 April 2007*).

In parallel with the process of seeking ethics approval from our institutional ethics committee, in March 2007, we contacted the National Co-ordinator of Ethics Committees, Ministry of Health (MoH), to determine the practicalities of retrieving the ethics applications from the MoH archives. Following several months of correspondence, we were asked to submit an application for expedited review by the Multi-Region Health and Disability Ethics Committee (MRHDEC). The MRHDEC reviews health and disability research proposals which are undertaken in more than one region (Figure 2). Expedited review is used to assess low-risk observational research or audit and related activity. These applications are reviewed by only the chairperson, or a delegated member of the Committee.

We submitted our application in November 2007. The chair of the MRHDEC considered the application and determined that since we were contacting trialists, the project needed to be considered by the full committee (December 2007). An application for review by the full Committee was submitted in January 2008. This was considered at the February meeting, where it received ethical approval, subject to providing two additional forms (locality assessment, evidence of consultation with an appropriate Maori group). Unconditional ethical approval was provided in March 2008.

We wrote to our institutional ethics committee in March 2008 advising them that we had received ethical approval to undertake the study from the MRHDEC, that we planned to proceed with the study, and asked them to raise any concerns with us. The Committee responded in May 2008 advising us that "The Committee would like to emphasise that they take no ethical responsibility for this study or the outcomes, considering their continued disapproval regarding the lack of informed consent.", followed by "The Committee acknowledges that the approval from the Regional Committee gives the researchers the authority to proceed, but that this approval does not equate to approval from University of Otago Human Ethics Committee." (*Personal communication: manager, Academic Committees, University of Otago, 5 May 2008*).

At this stage our head of department, who had been copied into much of the correspondence, wrote to our institutional ethics committee stating that he had read the ethics application and the NEAC guidelines for observational research [28] and was satisfied that the project was ethical and was happy for it to be based in the Department (*Personal communication: Head of Department, Department of Preventive and Social Medicine, University of Otago, 14 May 2008*). The Committee responded that they reaffirmed their position after examining the guidelines (*Personal communication: manager, Academic Committees, University of Otago, 10 July 2008*).

## Discussion

Participants of RCTs volunteer for many reasons. These include a belief that their participation may result in some improvement to their health, financial incentives, or altruistic reasons; such as the research they participate in resulting in benefits for others with the same condition in the future [21,30,31]. They do not participate to improve the financial status of pharmaceutical companies, nor do they participate to advance the career of academic researchers. In volunteering, they give their time and may experience inconvenience and financial loss. Moreover, there are potential harms associated with their participation. Trialists have a moral responsibility to the participants of their RCTs to conduct and report the results to the highest possible standard [21,32].

In recognition of substandard reporting practices, there have been many initiatives employed in an attempt to bring about improved reporting. These have included the development of reporting guidelines [24], voluntary registration of clinical trials, pre-registration of clinical trials as a condition of publication [33], the World Health Organization International Clinical Trials Registry Platform [34], journals accepting trial protocols for publication, the recently updated World Medical Association Declaration of Helsinki (principle 19) [35], and in the United States, legislation to make findings of clinical trials publicly available within a specific timeframe [36]. Some of these initiatives have contributed to an improvement in reporting practices [37] and this is likely to continue as greater requirements are placed on trialists to provide explicit information regarding outcomes and analytical methods prior to commencement of the trial. However, empirical research has still identified evidence of within-study selective reporting in cohorts of RCTs [15-17], some of which are relatively recent [15,17].

While internationally there is evidence of within-study selective reporting, we are unaware of any studies investigating this in NZ. An important step to improving the quality of reporting of RCTs is to assess current reporting against agreed standards of best practice so that processes can be developed to ensure adequate reporting if they are required. This approach is analogous to that used to improve quality in other sectors, including healthcare delivery and education. In these sectors performance measurement is used to determine areas for improvement, identify strategies with the potential to improve quality, and evaluate the impact of these strategies. As new evidence emerges to guide best practice, performance standards change, hence quality improvement is a continuous process.

In this publication we have considered the potential benefits and harms associated with undertaking the proposed research and have provided reasons why we believe the benefits out-weigh the harms. Other ethics committees, internationally, have come to the same conclusion and provided ethical approval for similar methodological research where retrospective access to ethics applications without informed consent from trialists has been sought [32]. However, our institutional ethics committee is not alone in its decision to not provide ethical approval for such research, with committees in Australia, the United Kingdom, and South Africa denying permission [32]. While some variation in ethics committees' decisions is expected due to the intrinsic nature of the committees, for methodological studies such as this, where there is public interest and little or no harm, the variation in decisions is intriguing and concerning. Reasons for this variation may include concerns about legal contractual obligations, litigation, and fear of upsetting multinational sponsers who may take their trials elsewhere. Variation may also exist from approaches the ethics committees use when considering ethical principles, and the level of importance they place on this type of methodological research. These two reasons are discussed further.

The reasons given for our institutional ethics committee's decision were largely based on the view that informed consent was a fundamental principle which permitted no exception. In taking this view the Committee was treating ethical principles as if they were legal rules. Yet principles are in conflict here, as is commonly the case in deliberation about the ethics of specific research projects. As well as autonomy, principles of beneficence, non-maleficence, and integrity are also relevant. This research had potential public benefit, not doing it may perpetuate a harm, and the whole undertaking supports the integrity of the research enterprise.

In law there are normally 'rules of recognition' that tell which rule prevails when they come into conflict. In ethics there are no agreed rules of recognition. Furthermore, deliberation simply at the level of rules or principles about research ethics overlooks the crucial detail of specific projects. Practical judgement is what is used in daily ethical decision-making and requires detailed knowledge of the facts of the case. Judgement also matters for research ethics. In a piece comparing the claims of ethical theory with practical judgement, Jonsen notes that "Justification of any particular moral claim comes rarely from a single principle ... but usually from a convergence of many considerations, each partly persuasive but together convincing with plausible probability" [38]. A central consideration for informed consent is what is being consented to. There is a huge difference between a clinical trial where participants may be asked to give their consent to receive a relatively untested intervention and consent to allow a document which contains no personal information to be examined.

In 2000, one of us (CP) reported on health researchers' views of ethics committee functioning and concluded that the quality of advice needed to be addressed: "The temptation to develop rigid rules to fit all circumstances can divert ethics committees from their chief function in which principles can be tested against the special circumstances of each case" [39]. Subsequently local guidelines designed to encourage deliberation of this sort for observational research, audits, and related activities, have been published [28]. However, our experience indicates that, at least for this ethics application, a rules-based approach is still evident.

The importance of this type of methodological research may not be valued or understood by members of ethics committees. In an editorial discussing a publication which investigated the effect of study design in RCTs on the magnitude of intervention effect, Hughes commented that "it is easy and may be tempting to overlook reports addressing methodologic issues in clinical trials and dismiss them to the niche market of professional statisticians and clinical academic researchers." [40]. Indeed, our institutional ethics committee was not convinced that the research was necessary. This was despite the fact that the research had been assessed by a panel of researchers for the purpose of determining whether it should receive a competitive internal university grant; which the panel deemed it should. Ethics committees must deal with scientific aspects that impinge on matters of potential harms and benefits. But it is questionable whether they should adjudicate on the worth of the research when scientific peers have approved it for funding. If committees are to undertake such a task, it would seem essential that the composition of the committee includes appropriate expertise to credibly assess the submitted applications [41].

The time between submitting the first ethics application and receiving ethical approval took seventeen months. The research project was funded for one year and with continued delays in receiving ethical approval, we ultimately were unable to use the funding to undertake the component of the project comparing ethics applications with their corresponding publication. Our experience is not unique, and if experiences such as this are not to be repeated in the future, mechanisms need to be developed to permit this type of research to be undertaken. While education of researchers about the importance of methodological research is one potential solution, more practical measures may be more effective. For example, prospectively informing trialists that their ethics application may be used in future methodological research or audits.

Alternatively, consideration could be given to changing the ethical review process to avoid conflict in decision making. It might help to have a separation between those who review proposals to undertake methodological research using ethics applications, and those with responsibility for reviewing ethics applications for RCTs. When there is no separation of these roles, this creates a potential conflict of interest. The ethics committee may regard the methods as ethical but be reluctant to approve it because of their relationship to the trialists and a possible, unwarranted, concern that identified failures of good reporting practice may implicate ethics committees themselves. In our case, although our institutional ethics committee did not have this dual role, it did suggest that such research would "more appropriately be undertaken as an audit by the relevant ethics committee", suggesting that it had somewhat of a conflict of interest; perhaps stemming from a concern of being included in future methodological research.

To address this sort of concern Chan *et al *[32] suggested a body, independent of the ethics committees, could be created which would consist of members who have expertise in ethics, public health, and the laws of privacy, contract, and intellectual property. This body would assess methodological research protocols and, if they were satisfied that the researcher's intention was to serve a greater public good while also undertaking to protect the privacy and proprietary rights of the applicants, could grant access to the ethics applications.

Regardless of the approach adopted for the ethical review of methodological research such as this, we concur with Chan *et al *that trial ethics applications should be treated confidentially and only aggregated data should be reported in resulting publications [32]. In our first application, and subsequent correspondence, we stated that our best efforts would be made not to allow identification of any particular study, and hence its investigators, but because of the relatively small number of studies, it could not be guaranteed that studies would not be identified by a small number people (e.g. other researchers working in the same area). We were, however, too blunt in our first ethics application where we stated that there was potential for embarrassment of trialists if it was pointed out that they may not have acted with complete scientific and ethical integrity and that this may not be a "bad thing".

We contend that a culture of continuous quality improvement is overdue in research and that the research community could benefit from a more explicit approach to quality improvement used in allied sectors. Improving quality in the conduct and reporting of RCTs is an ongoing process, driven in part by the findings of empirical methodological research and, in part, by the development of new reporting standards to address identified problems. Intuitively, improving quality should be a good fit with the research community since researchers are used to, and expect critical review of their research; this is an integral part of the academic process. A 2007 survey of researchers at a non-government research institute affiliated with a major children's hospital in Australia, exemplifies this with 79% (95%CI: 70%, 86%) reporting that they felt that auditing was an important part of the research process (response rate of 79%) [42]. Consistent with continuous quality improvement approaches in other sectors, the aim is to identify and address systemic causes of quality problems rather than apportioning blame to individuals.

Finally, there is a groundswell of researchers advocating for increased transparency of decision making processes by ethics committees and refuting concerns about the threat such transparency poses to researcher confidentiality and academic interests [43,44]. Greater transparency of decisions may confer many benefits. It may lead to greater protection of those who participate in clinical trials, improve the quality of the research, promote improved trust of ethics committees' decisions from the perspectives of researchers and the public, and provide researchers with opportunities to learn about the ethical review process. Importantly, a more open process of ethical review encourages the questioning of decisions and is consistent with the principles of quality improvement.

## Conclusion

Internationally, funders spend billions of dollars on RCTs annually. However, this investment is only a small fraction of what is spent on providing clinical treatments. High quality research is required to provide accurate information on which to base healthcare decisions. Funders and the public expect that research will be of the highest quality. To achieve this goal, research using ethics applications is essential. In NZ we have legislation which enables this, but we also require the support of the research community and those who sit on ethics committees. There are mounting calls for public access to ethics applications, full protocols and regulatory submissions [22,32,45]. We join these calls and hope that this publication promotes discussion of these issues.

## Abbreviations

CI: confidence interval; MoH: Ministry of Health; MRHDEC: Multi-Region Health and Disability Ethics Committee; NEAC: National Ethics Advisory Committee; NZ: New Zealand; NZRECs: New Zealand Regional Ethics Committees; RCT: randomised controlled trial.

## Competing interests

CP was a member of the NEAC from 2002 to 2007. GPH was a member of the Otago Ethics Committee from 1999 - 2004. JEM and PR declare they have no competing interests.

## Authors' contributions

JEM and GPH conceptualised and designed the "Conduct and reporting of RCTs in NZ" project, and applied for funding and ethics approval. They corresponded with the University of Otago Human Ethics Committee. CP and PR contributed their expertise to this correspondence but were not signatories of the correspondence, nor were they investigators of the project. JEM wrote the first draft of this publication excluding "What are the legal issues?" (Additional file 1) and text on the ethical issues in the discussion, which were contributed by PR and CP respectively. All authors contributed to revisions of the manuscript, have read and approved the final manuscript, and take public responsibility for its content.

## Authors' information

JEM and GPH are biostatisticians who are both authors of Cochrane systematic reviews of interventions. They both hold statistical editorial roles in The Cochrane Collaboration; an international organisation which produces and disseminates systematic reviews of healthcare interventions [46].

## Supplementary Material

Additional file 1 **What are the legal issues?**. An in-depth discussion of the legal issues surrounding access to ethics applications in NZ. Click here for file
