# Supplementary material for: Obstacles to researching the researchers: A case study of the ethical challenges of undertaking methodological research investigating the reporting of randomised controlled trials
Source: Trials. 2010 Mar 21;11:28. doi: 10.1186/1745-6215-11-28 (PMC2846843; doi:10.1186/1745-6215-11-28)
Supplement: Additional file 1 — What are the legal issues?. An in-depth discussion of the legal issues surrounding access to ethics applications in NZ. [file 1745-6215-11-28-S1.PDF]

## What are the legal issues?

The starting point in law is the Official Information Act 1982, which is New Zealand's principal freedom of information legislation. The legislation covers all "official information", which is defined as such depending on whether or not it is held by a public sector organisation. New Zealand Regional Ethics Committees fall under this definition. Indeed, it is the responsibility of ethics committees to inform trialists submitting ethics applications that the information they provide may be discoverable under the Official Information Act [1]. Whether trialists are informed of this fact or not, however, the ethics applications are still subject to the Act.

The long title to the Official Information Act expresses its principal purpose as "An Act to make official information more freely available". Its underlying public policy purpose is to promote the accountability of public sector agencies, actors and decision makers [2]. Section 4 sets out the "principle of availability" of official information. It provides that whenever an issue arises as to whether information should be made available or not, that issue must be determined "in accordance with the purposes of this Act and the principle that the information shall be made available unless there is good reason for withholding it." The Act specifies a limited number of "good reasons" that can be invoked by an agency for withholding information, and these form, as it were, a code. None of those "good reasons" applied to the ethics applications that were sought for this research project. Moreover, the Act's presumptions would clearly weigh in favour of disclosure of the ethics applications where public interests were involved; where public monies were to be expended; or where public sector employees, contractors, and decision making bodies (i.e., researchers and ethics committees) were subjected to scrutiny.

The Privacy Act 1993 governs the collection and availability of "personal information", which s 2 of the Act defines as "information about an identifiable individual". Information contained in ethics applications, as well as the questionnaire information proposed to be collected from trialists in this project, was undoubtedly largely information "about" the trialists, though it is arguable that taken in isolation, particular information could be construed to be about a research project rather than "personal information" about a trialist. As the Privacy Act and the Official Information Act are subject to each other, the two Acts need to be applied in a way that is mutually consistent. The Privacy Act was relevant to the handling of personal information in the research project in two ways.

Firstly, information privacy principle 11 in s 6 of the Act deals with the disclosure of personal information, and it provides that an agency may not disclose personal information unless the agency believes, on reasonable grounds, that one of the exceptions provided for in principle 11 applies. Disclosure arose as an issue because the proposed research project depended on the New Zealand Regional Ethics Committees disclosing the ethics applications they held. Principle 11(h) contained a clear exception that applied to this disclosure, in that the information concerned:

(i) Is to be used in a form in which the individual concerned is not identified; or
(ii) Is to be used for statistical or research purposes and will not be published in a form that could reasonably be expected to identify the individual concerned

Even if the information had been disclosed in breach of principle 11, this would not have necessarily breached the Privacy Act, since s 66 provides that in order to breach the Act there must be some loss or harm caused to the individual concerned by the breach, and in the case of emotional harm, the humiliation, loss of dignity or injury to feelings must be significant.

As noted in the publication, the University of Otago Human Ethics Committee (UOHEC) questioned "whether applications for ethical approval that have been submitted to an ethics committee are in the public domain." (*Personal communication: manager, Academic Committees, University of Otago, 19 January 2007*). Since the applications were held by a public sector organisation that is subject to the Official Information Act, and there was no Privacy Act reason for not disclosing them, they were publicly accessible as of right, and to that extent fell in the public domain. While the information was publicly accessible, the UOHEC essentially attempted to prevent them from being used for research purposes by declining the ethics application for the project.

Secondly, the Privacy Act was relevant because the proposed research project involved the collection of personal information in two respects: the collection of the trialists' research publications, and the collection of information directly from trialists through two questionnaires. Information privacy principles 2 and 3 provide that personal information must be collected directly from the individual concerned unless an exception applies (principle 2), and that where an agency collects personal information directly from an individual, the agency must take reasonable steps to make the individual aware of the nature of the collection of information and other related matters (principle 3).

The collection of the trialists' research publications was covered by both the principle 2(2)(a) and (g) exceptions. The principle 2(2)(a) exception provides that it is not necessary to collect information directly from the individual concerned if "the information is publicly available information", which is defined in s 2 as "personal information that is contained in a publicly available publication". The principle 2(2)(g) exception is in nearly identical terms to the principle 11(h) exception (above).

As for collecting information from trialists directly though the questionnaires, this is permissible if the matters required under principle 3(1) are communicated to the individual concerned: namely, the fact that personal information is being collected; the purpose for the collection of the information; the intended recipients of the information; the name and address of the agency that is collecting and that will hold the information; the consequences (if any) for the individual if the requested information is not provided; and notification of the individual's right of access to and correction of personal information. These requirements cumulatively amount to the soliciting of informed consent from the individuals concerned.

Furthermore, even if the researchers had failed to comply with principle 3, the collection of information would be permissible if it was reasonably believed that the information would be used in a form where the individuals concerned were not identified, or used for statistical or research purposes and published in a form that could not reasonably be expected to identify the individuals concerned.

That the UOHEC regarded the invocation of the Official Information Act and the Privacy Act as “unduly aggressive” in this context seems surprising, since the UOHEC had itself first raised the issue whether applications for ethical approval submitted to an ethics committee were in the public domain. It also seems odd that the citing of legal rights and obligations in response to the UOHEC’s query should be described not merely as “aggressive”, but as “unduly aggressive”. Pointing out to the UOHEC the public availability of ethics applications of approved randomised controlled trials under freedom of information legislation should surely have been highly relevant to any decision on the ethics of using them for research purposes. The Official Information Act imposes no limits on what use can be made of information obtained under the legislation.

The project’s ethics application was therefore caught in a strange stand-off where the law unambiguously provided for rights of access to and use of the information in question, but the UOHEC cited ethical considerations to defeat the responsible exercise of those legal rights, thereby thwarting the public policy reasons underlying the enactment of the legislation in the first place.

## References


1. Cook S: **Manual for chairpersons of health and disability ethics committees [http://www.ethicscommittees.health.govt.nz/].** 2004.
2. Committee on Official Information: **Towards Open Government: General Report.** (Wellington, 1980) vol 1: 14-15.
